# Food-Derived Tripeptide–Copper Self-Healing Hydrogel for Infected Wound Healing

**DOI:** 10.34133/bmr.0139

**Published:** 2025-02-03

**Authors:** Han Chen, Pu Yang, Ping Xue, Songjie Li, Xin Dan, Yang Li, Lanjie Lei, Xing Fan

**Affiliations:** ^1^ Department of Plastic and Reconstructive Surgery, Xijing Hospital, Fourth Military Medical University, Xi’an 710032, China.; ^2^ Department of Plastic and Aesthetic (Burn) Surgery, The Second Xiangya Hospital, Central South University, Changsha 410011, China.; ^3^ Key Laboratory of Artificial Organs and Computational Medicine in Zhejiang Province, Institute of Translational Medicine, Zhejiang Shuren University, Hangzhou 310015, China.

## Abstract

The field of infected wound management continues to face challenges, and traditional methods used to cope with wounds include debridement, gauze coverage, medication, and others. Currently, synthetic and natural biomaterials are readily available today, enabling the creation of new wound dressings that substantially enhance wound healing. Considerable attention is being paid to hydrogels based on natural materials, which have good biocompatibility and degradability properties, while exhibiting higher similarity to natural extracellular matrix as compared to synthetic materials. In this study, we extracted the active ingredients of oxidized konjac glucomannan (OKGM) and fresh egg white (EW) from 2 foods, konjac, and egg, respectively, and formed a self-repairing hydrogel based on the cross-linking of a Schiff base. Subsequently, a natural active peptide, glycyl-l-histidyl-l-lysine-Cu (GHK-Cu), was loaded, and an all-natural composite hydrogel dressing, EW/OKGM@GHK-Cu (GEK), was developed. The GEK hydrogel, exhibiting both antibacterial and anti-inflammatory properties, plays a hemostatic role by adhering to tissues and promoting neovascularization and serves as an optimal dressing for skin regeneration. Taken together, GEK hydrogel dressings derived from natural food sources therefore constitute an efficient and cost-effective strategy for managing infected wound healing and have significant potential for clinical application and transformation.

## Introduction

The skin plays a crucial role in maintaining basic body functions, including the protection of internal organs, removal of waste products through sweating, and the regulation of body temperature [1,2]. Trauma, surgery, and other injuries can lead to the loss of the skin barrier, for which conventional wound management strategies, such as gauze and antibiotics, are limited [3]. There has been considerable advancement in the development of biomaterials for tissue repair processes, such as tissue engineering scaffolds and nano-drug delivery, among which hydrogels are regarded as one of the most promising materials [4–7]. With the increasing demand for wound healing management and frequent dressing changes, the development of biologically active and cost-effective wound dressings with high healing capacity is urgently required. Hydrogel dressings are a prospective strategy for the treatment of skin injuries, as their excellent adherence and hydrophilicity properties allow them to adhere to wounds quickly while providing a moist microenvironment to facilitate wound healing and hemostasis [8–10]. Self-repairing hydrogels can form gels in situ and rapidly self-repair, enhancing their durability and reliability in real-world applications [11–13]. In addition, they can be loaded with bioactive molecules, making them an interactive dressing that can be rebuilt after injury and used in different types of injuries [14].

Hydrogels prepared from natural materials are more biocompatible and biodegradable than the synthetic ones [15,16], which prompted us to look into natural foods, namely, eggs and konjac tubers, in an attempt to develop a hydrogel dressing based entirely on natural materials. Egg white (EW) is a readily available, low-cost protein; each EW (excluding the yolk) contains approximately 3.6 g of pure protein, which is rich in amino acids and vitamins, making it a nutritious food [17,18]. Furthermore, EW is a time-honored ointment (used in ancient times) that forms a network of protein gels with remarkable wound healing potential [19]. It contains thousands of bioactive substances that promote cell proliferation, migration, differentiation, angiogenesis, and anti-inflammation, making it one of the most desirable raw materials for biomaterial applications [20,21]. Additionally, the EW solution is rich in oval transferrin and lysozyme, both of which possess antimicrobial activities [22]. The konjac tuber contains konjac glucomannan (KGM), a high-molecular-weight natural hydrophilic polysaccharide [23,24]. KGM is an anti-inflammatory, provascular, and immunomodulatory material used for wound healing; however, its use for that purpose has not yet received much attention [25,26]. Oxidatively modified konjac glucomannan (OKGM) is a natural candidate for hydrogel dressings owing to its good biocompatibility, water absorption, and gel-forming ability [27,28]. We developed a novel self-repairing hydrogel, OKGM/EW (EK) hydrogel, where the abundant aldehyde group in OKGM reacts with the amino groups of peptides and proteins in EW to form Schiff base bonds. It is a natural hydrogel based on food source, readily available and inexpensive with very good biocompatibility.

Loren Pickart, a biochemist, first isolated glycyl-l-histidyl-l-lysine (GHK) from the plasma in 1973 [29]. GHK is a tripeptide containing a particular amino acid sequence that can form a stable complex with copper ions, GHK-Cu [30]. GHK-Cu stimulates collagen and elastin formation, supports angiogenesis, and exhibits anti-inflammatory and antioxidant properties [31,32]. Moreover, it is cost-effective compared to other vasoactive peptides. This peptide is clinically approved and recognized for topical application as a skin rejuvenator [33]; however, it has rarely been used in natural hydrogels for wound repair. Being a natural peptide, GHK-Cu is prone to degradation, which hinders its clinical application. Nevertheless, loading it on hydrogels can result in slow drug release and effectively prevent the problem of rapid degradation [34,35]. This slow-release mechanism not only prolongs the duration of action of GHK-Cu but also ensures its sustained action in small doses, which is in line with the prerequisites for wound healing. Based on this concept, we innovatively integrated GHK-Cu into our prepared EK hydrogels via Schiff base reaction to verify its potential to promote skin healing.

**Fig. 1.**
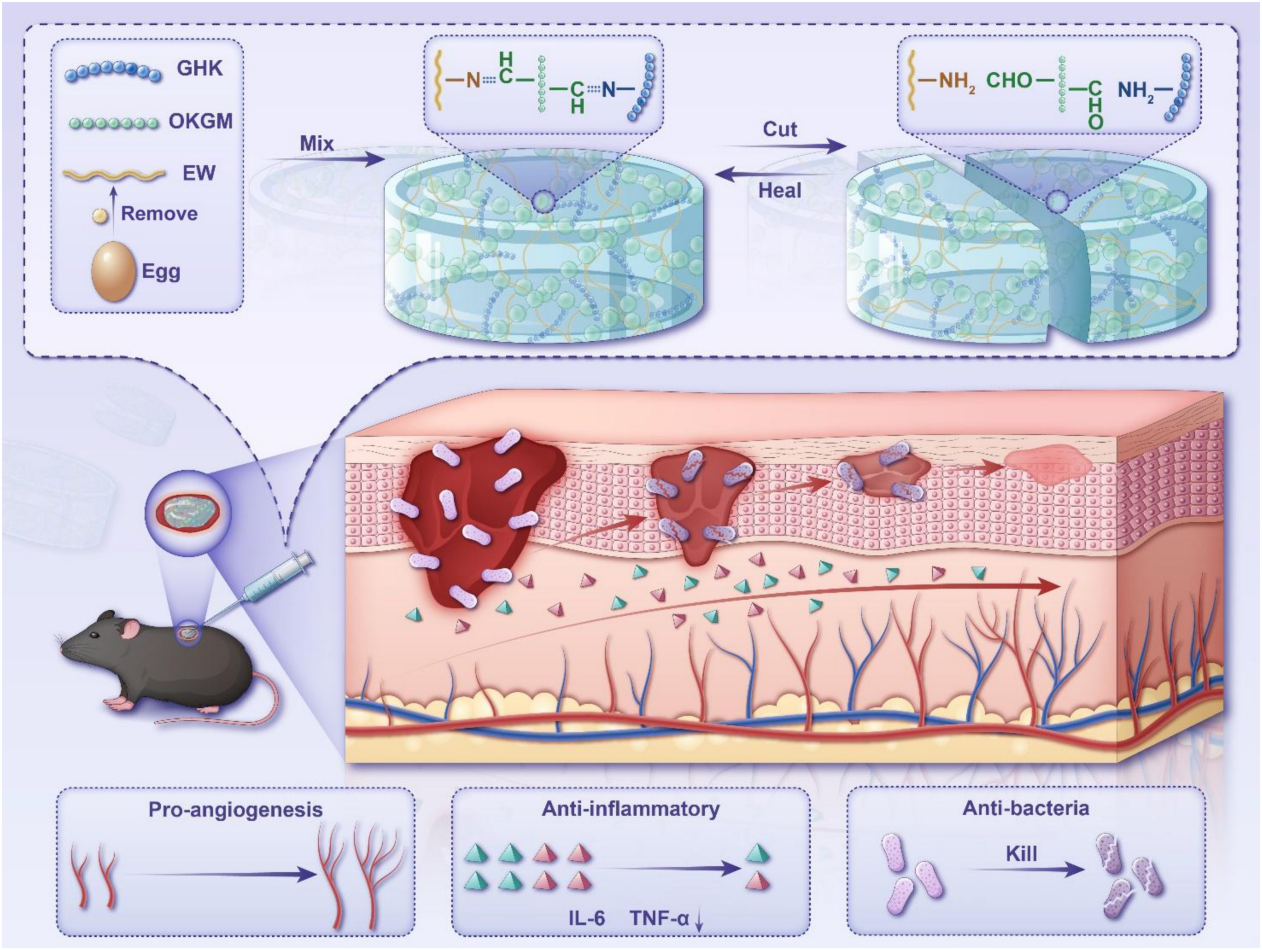
Schematic illustration of GEK hydrogel used in the repair of infected skin wounds.

In this study, we developed a natural, composite, multifunctional hydrogel dressing, EW/OKGM@GHK-Cu (GEK). The theoretical basis of this study is as follows: OKGM and EW were cross-linked (based on the Schiff base) to form a self-repairing EK hydrogel, and the amino group of active peptide GHK-Cu can also form Schiff base bond with the aldehyde group of OKGM. Furthermore, we evaluated the rheological properties and injection characteristics of the developed GEK hydrogels. In addition, we evaluated the feasibility of using the GEK hydrogel for the treatment of infected skin wounds by conducting several experiments, including in vitro experiments, tubulogenesis assay, cell migration assay, hematocompatibility assay, antimicrobial assay, and in vivo experiments, including the development of an infected wound mouse model and hemostasis assay. The experimental results showed that the developed multifunctional hydrogel is a promising option for wound healing applications. This study lays the foundation for leveraging the bioactivity of GEK natural hydrogels for wound healing, thereby presenting a new therapeutic strategy for skin healing (Fig. 1).

## Materials and Methods

### Materials

GHK-Cu was purchased from Shanghai Aladdin Biochemical Technology Co (Shanghai, China). KGM, sodium periodate, ethanol, and ethylene glycol were purchased from Macklin Biotechnology (Shanghai, China). The remaining reagents were of analytical grade and did not require additional purification. All aqueous solutions were prepared using ddH_2_O (Millipore, Billerica, MA). Hematoxylin and eosin (H&E) and Masson’s trichrome staining kits, Matrigel, and Calcein-AM/PI were bought from Servicebio Technology (Wuhan, China). Human keratinocyte cell line (HACAT) and human umbilical vein endothelial cells (HUVECs) were purchased from Procell Life Science&Technology and passaged for more than 3 generations. Luria–Bertani (LB) liquid medium and LB agar plates were bought from Biosharp. Cell culture media, including Dulbecco’s modified Eagle’s medium (DMEM; DMEM-F12) and phosphate-buffered saline (PBS) buffer, were purchased from ZETA life (USA), as was the cell counting kit-8 (CCK-8). Male C57BL/6 mice and male Balb/c mice (18 to 25 g, 8 weeks) were purchased from the Animal Experiment Center of the Air Force Military Medical University (Shaanxi, China). The Air Force Military Medical University Animal Experimentation Ethics Committee approved and reviewed all the animal care and experimental procedures (approval number: IACUC-20240003).

### Preparation of EW

We used a previously outlined method of isolation to obtain EW [36]. Pasteurized aseptic brown-shelled eggs (55 to 70 g) were purchased from a local food supermarket to ensure that the product was not spoiled. The EW was separated from the yolk, after which the viscous and nonviscous components were gently mixed and stored in a 4 °C refrigerator in an airtight container for later use, considering that their shelf life is only 1 week.

### Preparation of oxidized KGM

OKGM was prepared according to the literature with minor modifications [37]. First, 1 g of KGM was dissolved in 100 ml of ddH_2_O, heated, and stirred at 40 °C until completely dissolved, followed by the addition of 0.5 g of sodium periodate. After the reaction was executed for 4 h under light protection conditions, it was terminated by adding 1 ml of ethylene glycol. Subsequently, the dialysate was dialyzed in pure water with dialysis bags (molecular weight cutoff: 10 to 14 kDa) for 3 d. The dialysate was changed thrice a day until it was free of iodate. Finally, the dialysate was freeze-dried.

### Preparation of hydrogels

The prepared OKGM powder was added to PBS and stirred until completely dissolved, resulting in a final OKGM concentration of 3 wt %. Subsequently, we successfully prepared the composite GEK hydrogel using the following method: GHK-Cu was homogeneously mixed with EW solution and then with an equal volume of 3 wt % OKGM solution. The GEK hydrogel was obtained after complete cross-linking. GHK-Cu/OKGM (GK) hydrogels and EK hydrogels were obtained using the same methodology.

### Characterization of hydrogels

The infrared spectra of OKGM and KGM were acquired with a Fourier transform infrared spectrometer (FT-IR; Thermo Fisher Nocolet IS50). For the nuclear magnetic resonance (NMR) measurements (Bruker AVANCE NEO 600), we dissolved the fully dried OKGM powder in D_2_O until complete dissolution and then transferred it to an NMR tube using a disposable pipette. Subsequently, we conducted experiments on hydrogel formation and macroscopic injectability. The vial tilt method was used to determine the degree of gelation in the hydrogels. The flow of the sample was observed, and the gelation process was determined to be complete when the sample no longer flowed. An aliquot of 5 μl of red pigment (food grade) was added to the prepared GEK hydrogel, as the bright color is more suitable for observing the injectability of the hydrogel. A special “ZX” pattern was drawn with a 1-ml medical syringe to verify injectability. Next, we analyzed the topography and energy spectrum of the hydrogels. The microscopic morphology of EK, GK, and GEK hydrogels was observed via field-emission scanning electron microscopy (FE-SEM; QUTAN FEG 250, FEI) and analyzed using energy-dispersive spectroscopy (EDS). We tested the rheological properties of the GK, EK, and GEK hydrogels using a rheometer (Smartpave, Anton Paar). We carried out rheological experiments, including testing of shear thinning properties, dynamic time-scanning experiments, and structural recovery tests, on various groups of hydrogels.

Subsequently, we carried out swelling measurements of the hydrogels. Hydrogel samples were freeze-dried and weighed, immersed in PBS at 37 °C, analyzed, and then removed and weighed again. The swelling ratio (SR) was calculated as follows: SR = (*W*_a_ − *W*_b_)/*W*_b_ × 100%, where *W*_a_ denotes the actual weight of the hydrogel sample at a given time, and *W*_b_ denotes the initial weight of dry hydrogel.

We then analyzed the degradation characteristics of the hydrogels in detail. The fully crosslinked hydrogel samples were placed in PBS at 37 °C, and their weights were measured periodically until they completely degraded. The degradation rate was calculated as follows: Remaining weight (%) = (*W*_c_ − *W*_d_)/*W*_d_ × 100%, where *W*_c_ denotes the initial weight of the complete gel, and *W*_d_ indicates the weight of the gel at a specified time interval. Finally, with reference to previous literature [34], we tested the drug release of GHK-Cu in hydrogels with ultraviolet spectrophotometry in vitro.

### Cytocompatibility

The cytocompatibility of the different components of the hydrogels with HUVEC and HACAT was investigated using the CCK-8 assay. The leachate of hydrogels was obtained by completely sterilizing each group of hydrogels and immersing them in serum-free culture medium. Cells were inoculated in 96-well plates with 2 × 10^4^ cells, 10 μl of hydrogel exudate, and 90 μl of culture medium per well. Calcein-AM/PI staining was performed after incubation for 2 d at 37 °C, and the reaction mixture was incubated in the dark for 10 min. An inverted fluorescence microscope (Nikon, DS-Ri2, Japan) was used to obtain fluorescence images. The CCK-8 solution (10 μl) was added, and the absorbance value of CCK-8 at 450 nm was determined using an enzyme marker.

### In vitro angiogenesis

First, Matrigel was uniformly coated on a 96-well plate, and after it solidified, each well was inoculated with 2 × 10^4^ HUVEC. The experiment was divided into 4 groups; PBS was added to the control group solution, whereas 100 μl of the hydrogel exudate was added to the solutions of the other groups. After incubation for 10 h, the tubules were stained with Calcein-AM for 20 min. After being washed with PBS, the cells were observed. Angiogenesis was analyzed with ImageJ to determine the relative tube lengths. This experiment was repeated at least thrice.

### Cell scratch migration assay

HUVECs were resuspended with DMEM-F12 medium and inoculated in 6-well plates with a density of 1 × 10^6^ cells per well. After cell monolayer formation, a sterile 100-μl pipette tip was used to slowly draw a line on the culture plate. Hydrogel exudates from different groups were co-incubated with HUVEC. The width of the scratches in each group was observed and recorded under an optical microscope; the images were acquired again after 48 h of incubation at 37 °C. The migration pattern of HUVEC on the culture plate was observed, and the widths of the scratch before and after migration were compared among the groups using ImageJ. The migration ability of the cells was evaluated by calculating the migration rate.

### In vitro hemolytic assay

The hemocompatibility of GK, EK, and GEK hydrogels was assessed by measuring their hemolytic activity. Mouse whole blood was collected in heparin-containing anticoagulant tubes. Whole blood was washed repeatedly with PBS until the supernatant was clear after centrifugation. Subsequently, the erythrocyte precipitate was diluted with PBS, and then H_2_O (positive control), PBS (negative control), GK, EK, and GEK hydrogel exudates were incubated with the diluted erythrocyte solution for 2 h at 37 °C. The tubes were finally centrifuged at 3,500 rpm for 5 min to determine the extent of hemolysis in each group. The degree of hemolysis was assessed using an enzyme marker at 540 nm.

### In vitro antimicrobial measurements

The bacteriostatic properties of the hydrogels were determined using the plate counting method. Representative strains of *Staphylococcus aureus* and *Escherichia coli* were used for their antimicrobial experiments. First, an appropriate amount of *E. coli* and *S. aureus* strains was transferred to LB liquid medium and incubated for 12 h. The diluted bacterial suspensions were then mixed with 200 μl of PBS, GK, EK, or GEK hydrogel. After 10 h of coculture at 37 °C, the bacteria were evenly spread on LB agar plates, and the plates were incubated at 37 °C for 12 h. Bacteria in each group of the LB agar plates were quantified. The bacterial suspensions from each group after co-incubation were aspirated into 6-well plates. We inoculated silicon wafers (10 × 10 mm^2^) with bacterial suspensions for >12 h at 37 °C, after which they were incubated with bacteria from multiple strains. The silicon wafers were fixed with 2.5% glutaraldehyde for 4 h. Gradient dehydration with ethanol was then performed. Finally, the morphology of the bacteria was observed using SEM.

### In vivo hemostasis test

A mouse tail amputation model and a mouse hemorrhagic liver model were used to evaluate the hemostatic effect of the GEK hydrogel.

Briefly, 3 Balb/c mice were selected from each group, anesthetized with 1.5% isoflurane, and had their tail lengths clipped by 40%. Subsequently, the tails of the mice in the experimental group were covered with 150 μl of the GEK hydrogel, and the hemorrhage during hemostasis was recorded for 1 min. The control group was set as the untreated trauma group. The mouse hemorrhagic liver model was established as follows: Balb/c mice were anesthetized and then fixed on the mouse operating bed; the livers of the mice were exposed through an abdominal incision; the blood was collected by placing a filter paper under the liver. A 5-mm-deep hemorrhagic wound was inflicted in the liver of a Balb/c mouse, and 500 μl of GEK hydrogel was immediately applied to the hemorrhagic site. The control group was set up as the untreated group, and 6 animals were included in each group. The filter paper was weighed both before and after the experiment, and the blood volume was calculated.

### Infected wound mouse models

Twenty-four male C57BL/6 mice (18 to 25 g) were selected for this study. The mice were randomly divided into 4 groups comprising 6 mice each. An infected wound model of total skin injury was constructed to verify the effects of GK, EK, and GEK hydrogels on the repair of infected wounds. Specifically, the backs of the mice were first depilated with an animal depilatory cream to expose the extent of the trauma. The mice were then anesthetized via the inhalation of 1.5% isoflurane using an anesthetic respirator using a mask. A 10-mm-diameter full-layer skin wound defect was made on the back of each mouse, in which 100 μl of *S. aureus* suspension was injected to form an infected wound, followed by the application of Tegaderm film (3M, USA). After 24 h, the wounds were found to be red and swollen with purulent secretion, which meant that the model was created successfully. Subsequently, 100 μl of PBS, GK, EK, and GEK hydrogels was used to cover the skin wounds of mice. Images of the dorsal wounds of mice were captured on days 0, 3, 6, 9, and 12 using a digital camera, and the data were analyzed using ImageJ. After euthanizing the mice on day 12, periwound skin tissue was collected from different groups for follow-up experiments, as detailed in the subsequent section.

### Histological, immunohistochemical, and fluorescence staining

After euthanizing the animals, skin lesions without marginal tissue were removed and fixed in a paraformaldehyde fixative (Servicebio) for 12 h. Skin samples were first paraffin-embedded and then dehydrated with an ethanol gradient before being cut into 5-mm-thick sections. Sections were stained with H&E to observe the process of epithelialization during wound healing and assess the healing effect. Masson’s trichrome staining was performed on the sections to observe collagen deposition. Additionally, interleukin-6 (IL-6) and tumor necrosis factor-α (TNF-α) immunohistochemical staining was performed to evaluate the inflammatory responses of the infected wounds. Finally, CD31 and α-SMA (smooth muscle actin) fluorescence staining was used to evaluate the formation of neovascularization in the trauma.

### Statistical analysis

All experimental data are expressed as mean ± standard deviation (*n* ≥ 3) and were analyzed using Student’s *t* test to determine the statistical differences between 2 groups. In this study, asterisks (*) denote the statistically significant differences when comparing various experimental groups with the control group. All graphics were plotted using the Origin Pro 2021 software program (Origin Lab, Northampton, MA, USA), and differences were considered significant at *P* < 0.05 (*), *P* < 0.01 (**), and *P* < 0.001 (***).

## Results and Discussion

### Characterization of hydrogels

The EK, GK, and GEK hydrogels were cross-linked via the Schiff base reaction, which requires the KGM to be oxidized to form OKGM. The successful oxidation of KGM for hydrogel crosslinking was confirmed by characterizing the powders of KGM and OKGM using FT-IR spectroscopy. The peak at 1,745 cm^−1^ on the FT-IR spectrum of OKGM corresponds to the C–O stretching vibration of the acetyl group; this indicates the presence of carbonyl groups, which is consistent with the results reported in the literature (Fig. S1) [17]. The NMR spectrum of OKGM was recorded to characterize the compound by identifying its characteristic peaks; the results were consistent with previous research [38]. The aldehyde group of OKGM behaves as an enol under alkaline conditions, and the chemical substitution peaks at 9.24 and 6.49 ppm (parts per million) represent active hydrogen on the enol carbon. The experimental results confirmed the important structural features of OKGM (Fig. S2).

**Fig. 2.**
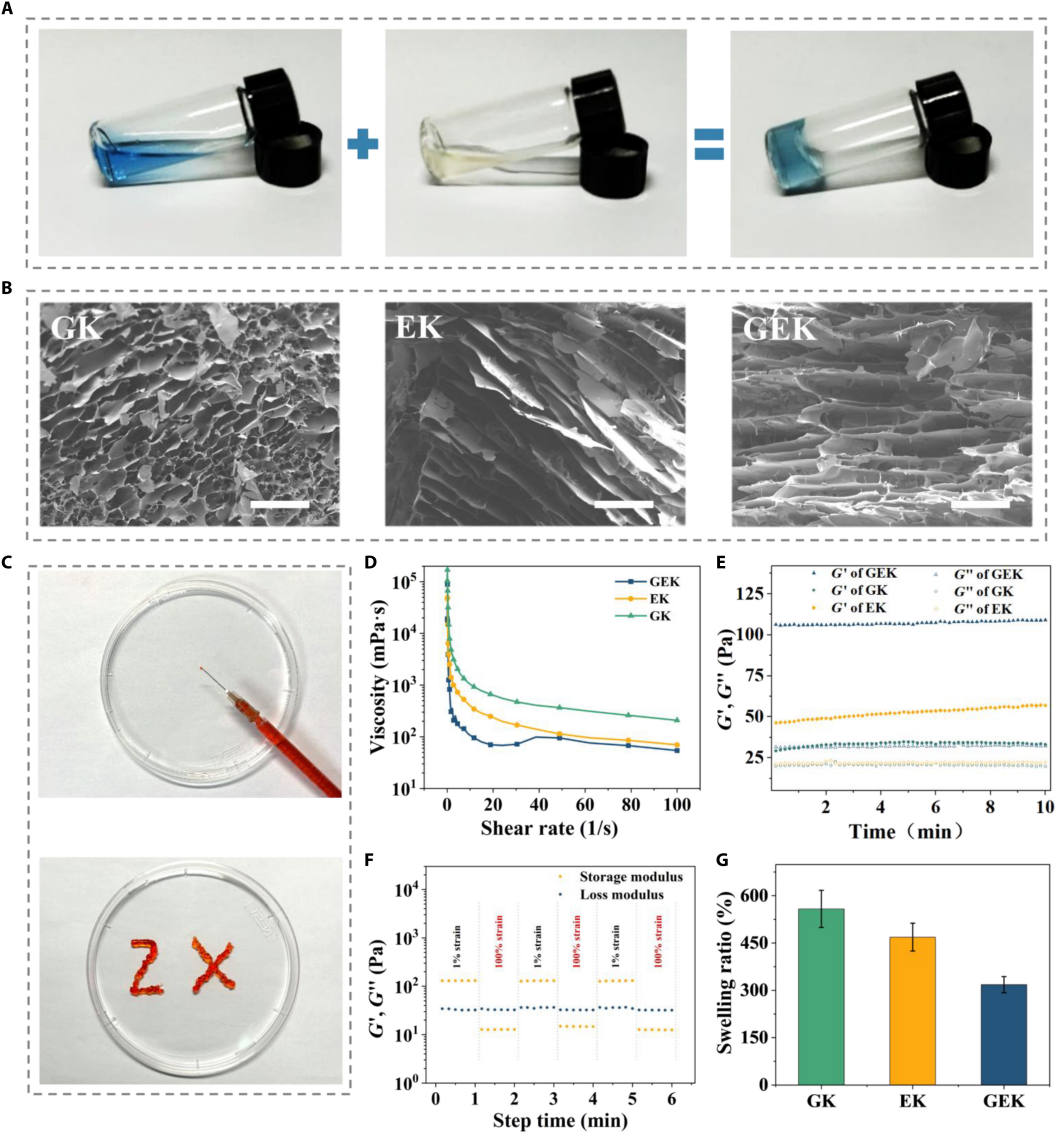
Characterization of hydrogels. (A) Formation of GEK hydrogel by mixing EW and OKGM loaded with GHK-Cu. (B) SEM image. Scale bar, 200 μm. (C) Macroscopic injectivity of GEK hydrogel. (D) Shear-thinning properties. (E) Dynamic rheology test. (F) Strain scanning test of GEK hydrogel. (G) Swelling properties.

Rapid gelation makes the dressing less likely to flow, resulting in sustained and effective wound treatment that is more compatible with practical applications. GEK hydrogel can spontaneously gel at 25 °C without intervention, as demonstrated by vial decantation experiments (Fig. 2A). In addition, hydrogels with microporous structures have good hydrophilicity, which facilitates moistening of the wound surface, absorption of wound exudate, and exchange of gases with the external environment. The morphology and pore size of the GK, EK, and GEK hydrogels were directly observed using SEM; the images showed that the 3 hydrogels formed typical microporous structures, which is ideal for skin regeneration (Fig. 2B). Elements of GEK hydrogel, including C, N, O, and Cu, appear in the EDS pattern (Fig. S3). The GEK hydrogel exhibited macroscopic injectability for direct application to the wound, which enabled the pattern “ZX” to be written continuously through a syringe without blockage when stained with red pigment (Fig. 2C).

The rheological properties of hydrogels are important indicators of the deformation and flow behavior during stress, including shear-thinning and microscopic gelation time, as well as self-repairing properties, which are critical for application in skin trauma. In practical applications, the injectability of hydrogels is an essential prerequisite. Therefore, the shear-thinning properties of each hydrogel group were evaluated. At a constant strain of 1%, the viscosity values of the EK, GK, and GEK hydrogels decreased as the shear rate increased, which indicated good injectability (Fig. 2D). We then performed frequency scanning tests, which showed that the *G*′ values of the EK, GK, and GEK hydrogels were all consistently greater than the corresponding *G*″, meaning that all groups were successfully gelated. The GEK hydrogels had the highest *G*′ values—approximately 106 Pa (Fig. 2E). Finally, we implemented low/high-strain-scanning cycles to test the self-repairing properties of GEK hydrogel networks. After 6 cycles, these moduli remained constant, which reflects the self-healing and stable mechanical properties of the hydrogel structures, the favorability of their application to irregular wounds (Fig. 2F). Next, we evaluated the hydrophilicity and solubility of GK, EK, and GEK hydrogels in PBS and found that each hydrogel exhibited good solubility. The weights of GEK hydrogels after swelling were 3 to 4 times higher than their original weights and exhibited the lowest equilibrium expansion ratio (ESR), which could be attributed to the highest degree of cross-linking (Fig. 2G).

Degradability is an important parameter for wound dressings, as hydrogels need to be degraded in a timely manner so as not to interfere with drug release owing to the short healing cycle of skin wounds. Different groups of GK, EK, and GEK hydrogels were able to degrade at 16 h; the remaining weight of the hydrogels was approximately 22% (Fig. S4). In vitro drug release experiments showed that GHK-Cu loaded into hydrogels were continuously released in PBS at 37 °C. In the early stage, the active peptide release rate was high and the release stabilized over time. At 96 h, the cumulative drug release rate of GHK-Cu from the hydrogel was approximately 62% (Fig. S5).

### Biocompatibility of EW/OKGM@GHK

**Fig. 3.**
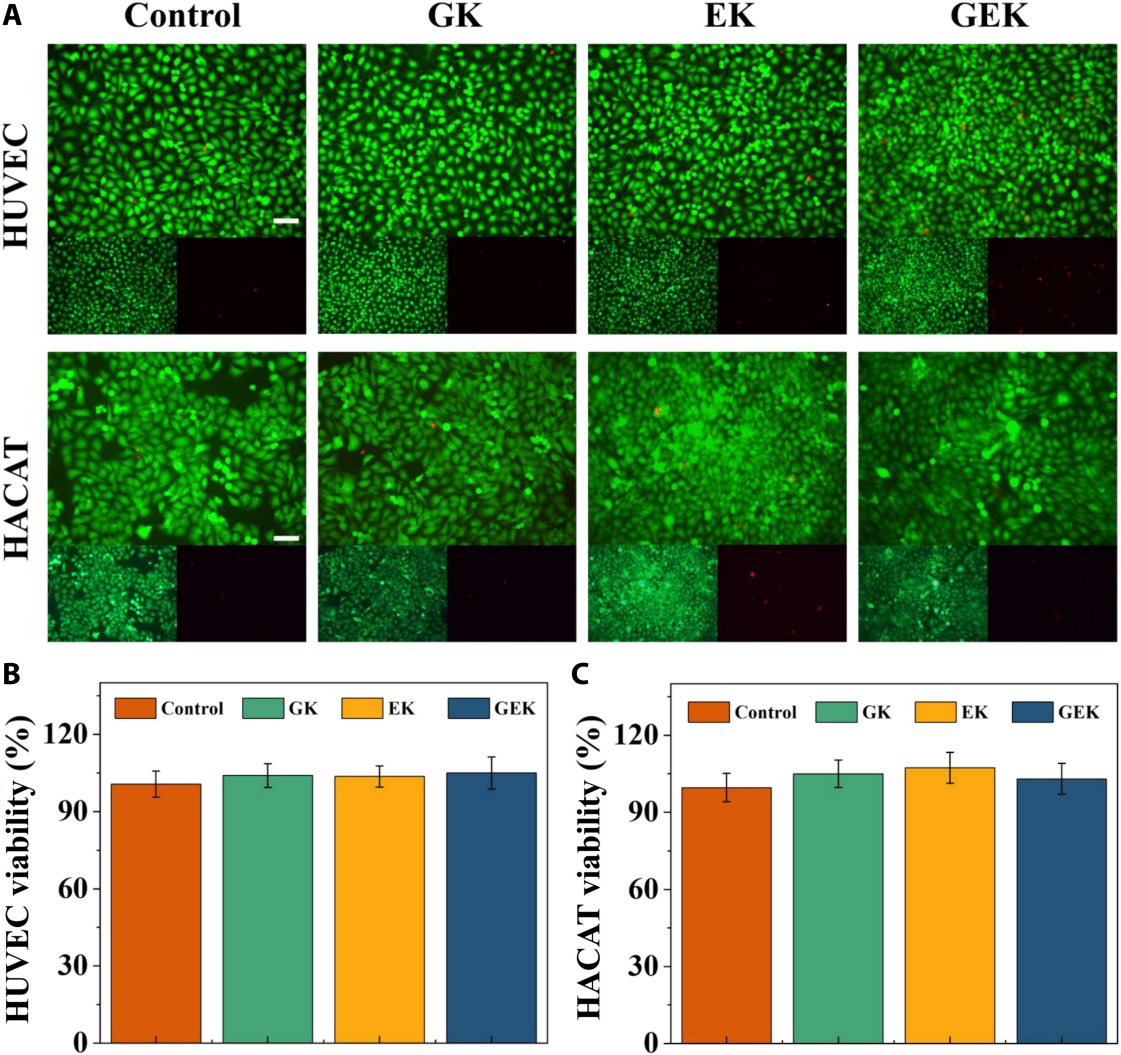
Cell biocompatibility. (A) Live/dead staining images of HUVEC and HACAT cocultured with hydrogel for 24 h. Scale bar, 100 μm. Quantitative analysis of the viability of (B) HUVEC and (C) HACAT.

Biocompatibility is a key property to consider when designing biomaterials and wound dressings. We applied the following methods and strategies to determine the biocompatibility of hydrogels to ensure the absence of cytotoxicity. Theoretically, the hydrogels crosslinked by the 3 natural biomaterials—OKGM, EW, and GHK-Cu—should have good biocompatibility, which still needs to be verified in specific applications. First, cellular-level validation was performed, and the cytotoxicity of the hydrogels was assessed by measuring their survival after co-incubation with HUVEC and HACAT using the CCK-8 assay. Results showed that the cells survived in all groups, i.e., the hydrogels were not cytotoxic to HUVEC. There was a significant increase in the number of HACAT cells in the EK and GEK groups on day 3, which could be attributed to the release of the hydrogel-loaded active ingredient that promoted cell proliferation (Fig. 3). Assessing the hemocompatibility of biomaterials before subcutaneous application is a critical step in verifying biocompatibility. We used heparinized blood collection tubes to obtain blood from the ophthalmic artery of Balb/c mice and evaluated the blood compatibility of the hydrogels using the hemolysis test. Results indicated that the prepared GK, EK, and GEK hydrogels had good hemocompatibility (Fig. S6). Finally, we performed organ compatibility experiments in animals in vivo, where we subcutaneously implanted 500 μl of GEK hydrogel into Balb/c mice. The heart, liver, spleen, lung, and kidney tissues were obtained 15 d later to assess potential organ toxicity. H&E staining results showed that there was no difference in the visceral tissue sections of the different groups (Fig. S7). In conclusion, GEK hydrogels can be safely used on acutely infected wounds.

### In vitro proangiogenic and prohealing activities of the hydrogels

**Fig. 4.**
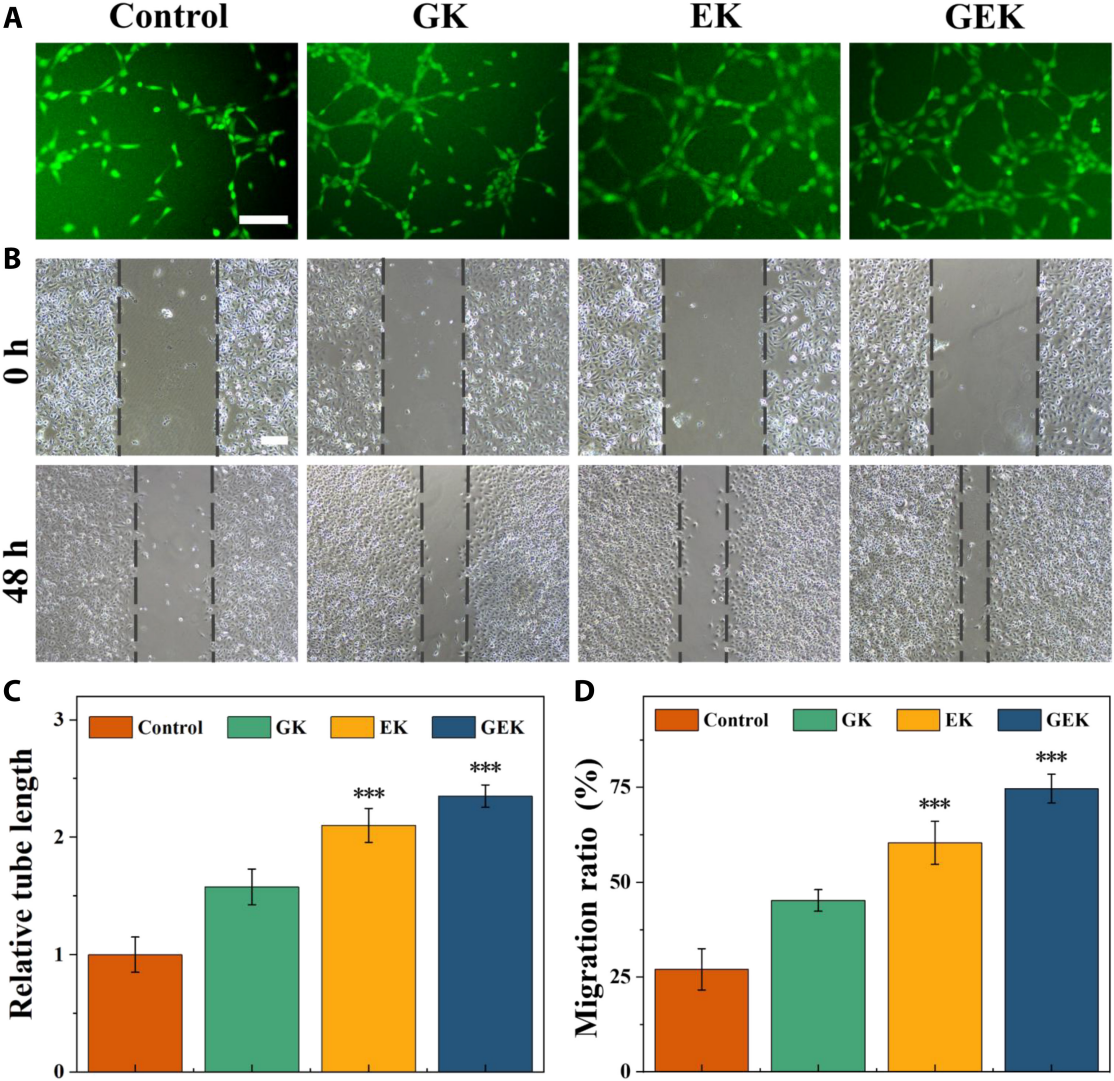
Tubular formation experiment and scratch experiment. (A) Formation of HUVEC tubes in different hydrogel treatment groups. (B) HUVEC migration in different hydrogel treatment groups. (C) Relative tube length of HUVEC. (D) Migration rate of HUVEC. Scale bar, 100 μm.

Accelerated wound vascularization is one of the key strategies used to promote wound healing, and neovascularization facilitates nutrient and metabolite exchange to promote wound healing. We assessed the ability of HUVEC to form tubular structures in GK, EK, and GEK using the tube-forming assay. The results showed that cells exposed to GEK and EK formed blood vessels more efficiently than did the cells from other groups (Fig. 4). We then investigated the promigration ability of GK, EK, and GEK hydrogels based on scratch experiments. The results showed that the GEK hydrogel significantly enhanced the migration of HUVEC within 48 h with an approximate 60.4% scratch closure rate as compared with 29.1% for that in the control group. The EK hydrogel also enhanced the promigratory ability of cells; this may be due to its high-ovalbumin content that improves cell adhesion and proliferation. GHK-Cu is remarkably biocompatible and has been extensively used to enhance cell adhesion. Accordingly, the incorporation of GHK-Cu into EK hydrogels further enhanced their cell affinity. Hydrogels may promote cell migration owing to this synergistic effect. Therefore, the GEK hydrogel effectively stimulated vascularization formation and induced cell migration.

### Antimicrobial properties of hydrogels

**Fig. 5.**
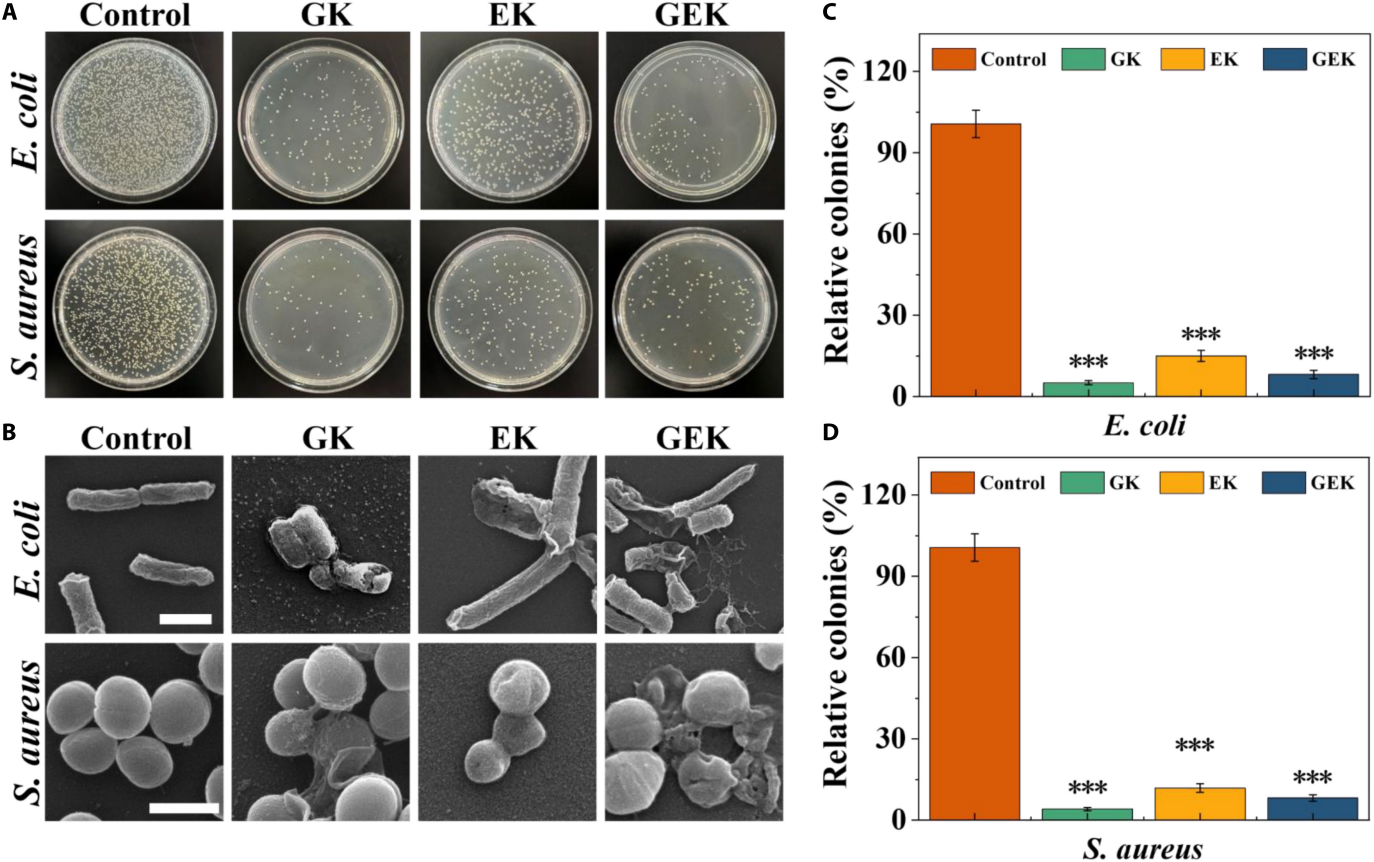
Antibacterial properties. (A) Antibacterial properties of each hydrogel group were tested using a bacterial coating. (B) SEM images of bacteria under hydrogel intervention. Scale bar, 1 μm. Quantitative analysis of the antimicrobial effect of hydrogels against (C) *E. coli* and (D) *S. aureus*.

To treat infected skin wounds, hydrogels with antimicrobial properties are necessary because skin wound infections disrupt wound healing. Taking *S. aureus* and *E. coli* as representatives of gram-positive and gram-negative bacteria, respectively, we performed cocultures of EK, GK, and GEK hydrogels. Bacteria-coated plate experiments showed that the hydrogels exhibited favorable antimicrobial properties, as there was a significant reduction in the number of bacterial colonies in the hydrogel groups after treatment (Fig. 5). Among them, the GEK hydrogel exhibited a stronger antimicrobial effect, i.e., infected skin wounds were better suited to its application. We collected the treated bacteria, fixed them, dehydrated them, sprayed the surface with gold, and observed their micromorphology using SEM. Compared with the control, EK, GK and GEK all caused *S. aureus* and *E. coli* to contract and rupture; these changes altered their physiological morphologies. This indicates that the prepared hydrogel is effective in killing both *S. aureus* and *E. coli*, which is mainly related to the release of ovaltransferrin, antimicrobial peptides, and GHK-Cu components by the hydrogel system.

### In vitro hemostasis test of the hydrogels

Hydrogels are effective in controlling bleeding, which can create a stable environment for wound healing. To evaluate the hemostatic potential of the GEK hydrogel, we chose typical hemostatic models, namely, the mouse tail amputation and liver hemostasis models. Results showed that the GEK hydrogel sealed the wound and stopped bleeding quickly, in contrast to that in the untreated tail/liver, which produced large areas of blood on the filter paper. In the GEK group, blood loss was 3 to 4 times lower than that in the control group, demonstrating good hemostatic effects in both the animal hemostatic models (Fig. S8). The hemostatic properties of the GEK hydrogel may be partially attributed to the tissue adhesion properties of the gel, allowing it to stably bind at the wound. In addition, OKGM enhances adhesion to other molecules, and the EW possesses hemostatic efficacy.

### Histological and in vivo wound healing analyses

To further evaluate the effect of the hydrogel in promoting the healing of infected wounds in vivo, we randomly divided mice into different groups, established wound models, applied different hydrogels, and monitored healing for 12 d. We euthanized the mice via cervical dislocation on day 12 and sampled the periwound skin tissue for subsequent experiments.

**Fig. 6.**
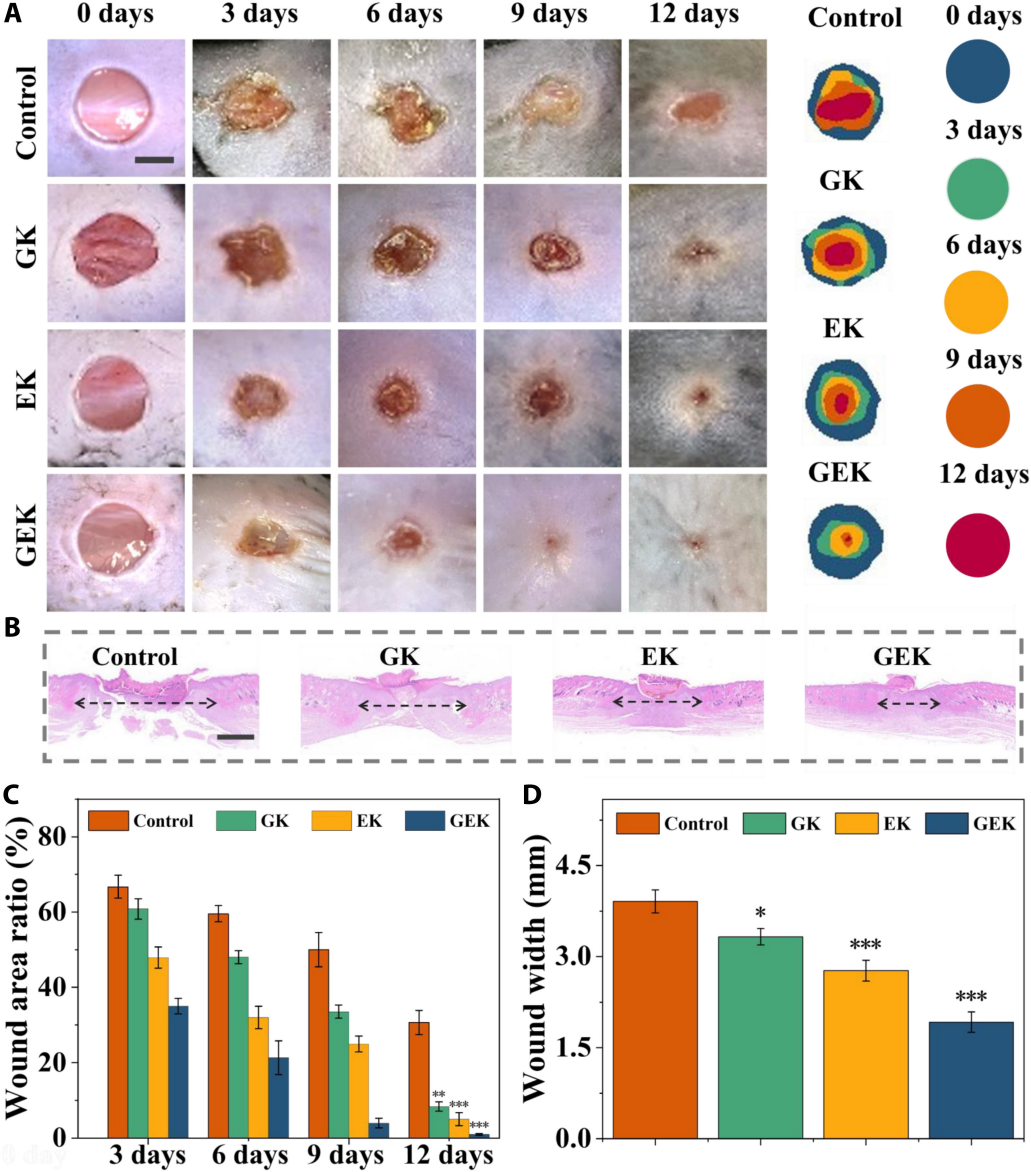
Wound healing and histological analyses. (A) Representative wounds. Scale bar, 5 mm. (B) H&E staining. Scale bar, 1 mm. Quantitative analysis of (C) wound healing and (D) tissue width.

A quantitative analysis of wound size showed that the GEK hydrogel group completed wound closure by day 12 with a healing rate of >95%, which is higher than that of the control group (~65%) (Fig. 6). The GK, EK, and GEK hydrogels were all capable of promoting wound healing, with increased healing areas compared with those in the control group. We used H&E staining to assess the histological changes in the wounds. Results showed that the control group had the highest degree of epidermal incompleteness, while the wounds in the hydrogel groups showed varying degrees of epidermalization. The GEK group had a smaller wound width and more complete wound healing patterns than those in the other groups, suggesting that it had enhanced epithelialization during wound recovery. Accordingly, we hypothesized that the sustained release of active substances in GHK-Cu and EW at low doses is more appropriate for wound healing. The GEK hydrogel may exhibit better efficacy in healing chronic wounds.

### Hydrogel-induced collagen deposition in wounds and inflammatory response in vivo

During wound healing and scar formation, collagen contributes to the extracellular matrix of the skin. We evaluated the degree of wound healing by assessing collagen deposition in skin tissues using Masson’s staining. Results showed that the hydrogel treatment resulted in the deposition of more collagen fibers in the dermis than did the control treatment, under which only a small amount was deposited in the control tissue. In addition, collagen deposition was the highest in the GEK group. Collagen deposition in the GK group was also higher than that in the control group but lower than that in the EK and GEK groups.

**Fig. 7.**
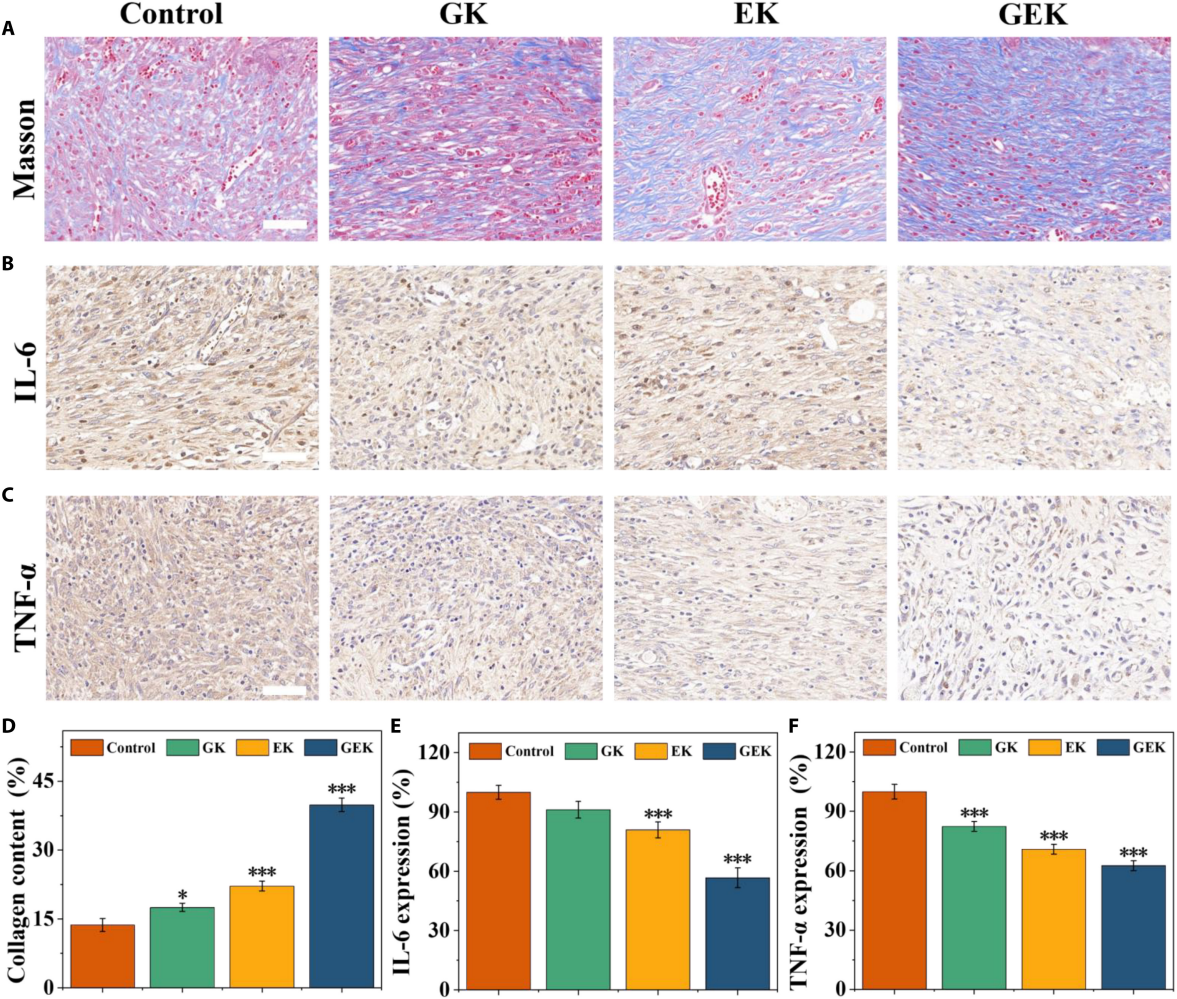
Analysis of collagen deposition and proinflammatory factors in wounds. (A) Collagen, (B) TNF-α, and (C) IL-6 staining. (D to F) Collagen deposition and relative expression of IL-6 and TNF-α. Scale bar, 50 μm.

The inflammatory milieu allows the determination of whether a normal injury will turn into a long-term or chronic wound and it is involved in the host’s defense against environmental pathogens. Sustained high IL-6 levels impair wound healing. The small-molecule protein TNF-α secreted by macrophages is a common indicator of inflammation and promotes the proliferation of immune cells. Immunohistochemical staining revealed that the GEK hydrogel caused a significant reduction in the expression levels of proinflammatory cytokines IL-6 and TNF-α as compared with the control treatment. These findings suggest that the GEK hydrogel can effectively regulate traumatic inflammatory activities (Fig. 7). The other hydrogel groups also showed reduction as compared with the control group. These results suggest that the developed GEK hydrogel can promote wound healing by increasing collagen deposition in infected wounds and reducing the inflammatory response.

### In vivo analysis of hydrogel-induced proangiogenesis

**Fig. 8.**
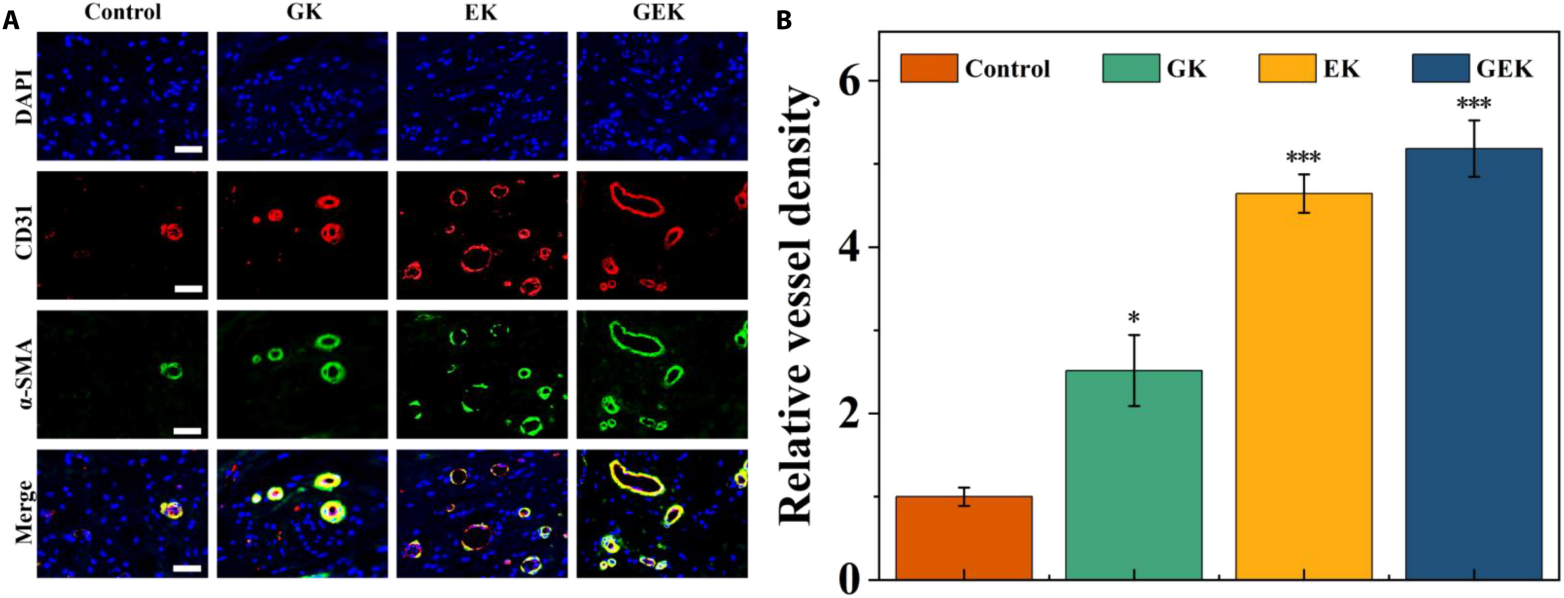
Immunofluorescent staining. (A) Fluorescent staining for neovascularization (α-SMA and CD31). Scale bar, 50 μm. (B) Relative vessel density.

Neovascularization in the vicinity of the wound is essential for wound healing. This stems from the fact that the formation of new capillaries increases blood perfusion to the wound, provides nutrients, and removes metabolites. To evaluate the proangiogenic activity of the composite hydrogel on wounds, we assessed neovascularization via the immunofluorescence staining of CD31 and α-SMA common indicators. We observed a significant increase in the density and extent of neovascularization and greater vascular regeneration in the GEK hydrogel (Fig. 8). This may be because the GEK group was able to not only slowly release the active substances of EW (tryptophan, l-arginine) but also release high amounts of GHK-Cu with vascular activity, which is consistent with the results of the precellular experiments.

## Conclusion

Damaged wounds often require external intervention, which recently triggered the development of multifunctional hydrogels for rapid healing. Hydrogel wound dressings not only are water and oxygen-permeable but also act as physical barriers to protect the wound from microorganisms. Accordingly, we prepared a multifunctional self-repairing GEK hydrogel based on a Schiff base reaction that promoted rapid healing of infected skin wounds. We found that the self-healing GEK hydrogel rapidly stopped bleeding, inhibited bacterial proliferation, modulated inflammatory response, and promoted vascular regeneration and epithelialization in wounds and the healing of infected wounds in mice. Moreover, this hydrogel exhibited favorable injection, swelling, and self-healing properties, which facilitated its convenient application to skin wounds. In particular, self-repairing hydrogels from natural sources have better biocompatibility and require mild reaction conditions when compared with synthetic compounds, thereby offering a higher potential for clinical translation. We innovatively introduced GEK-Cu into the hydrogel, which was released at a low concentration and was better suited to the physiological healing process. The EW is also a cost-effective raw material, which is more appropriate and worthy for commercialization. OKGM, as a modified polysaccharide with excellent biocompatibility and gel properties, enhanced interaction with cells, improved gel stability, and, most importantly, facilitated the slow release of the active ingredients from the EW and GEK-Cu. Despite these promising results, this study has certain limitations owing to the lack of a more in-depth exploration of the healing mechanisms. Moreover, validation related to medium-sized to large experimental animals is lacking and warrants further exploration. In the future, GEK hydrogels could be further explored for translational clinical applications, tissue engineering scaffolds, e-skin, drug delivery, and other applications, which provides a new dimension to the application potential of GEK hydrogels.

## Data Availability

Data will be made available on request.
