# Supplementary material for: Food-Derived Tripeptide–Copper Self-Healing Hydrogel for Infected Wound Healing
Source: Biomater Res. 2025 Feb 3;29:0139. doi: 10.34133/bmr.0139 (PMC11788471; doi:10.34133/bmr.0139)
Supplement: Supplementary 1 — Figs. S1 to S8 [file bmr.0139.f1.docx]

Title

**Food-derived tripeptide–copper self-healing hydrogel for infected wound healing**

**Authors**

Han Chen^1†^, Pu Yang^1,2†^, Ping Xue^1^, Songjie Li^1^, Xin Dan^1^, Yang Li^1*^, Lanjie Lei^3*^, Xing Fan^1*^

**Affiliations**

1. Department of Plastic and Reconstructive Surgery, Xijing Hospital, Fourth Military Medical University, Xi’an, 710032, China.
2. Department of Plastic and Aesthetic (Burn) Surgery, The Second Xiangya Hospital, Central South University, Changsha, 410011, China.
3. Key Laboratory of Artificial Organs and Computational Medicine in Zhejiang Province, Institute of Translational Medicine, Zhejiang Shuren University, Hangzhou, 310015, China.

Corresponding author E-mail:

Yang Li: liyangzx@fmmu.edu.cn

Lanjie Lei: leilanjie1988@163.com

Xing Fan: fanxing.612@163.com

^†^These authors contributed equally to this work.

**
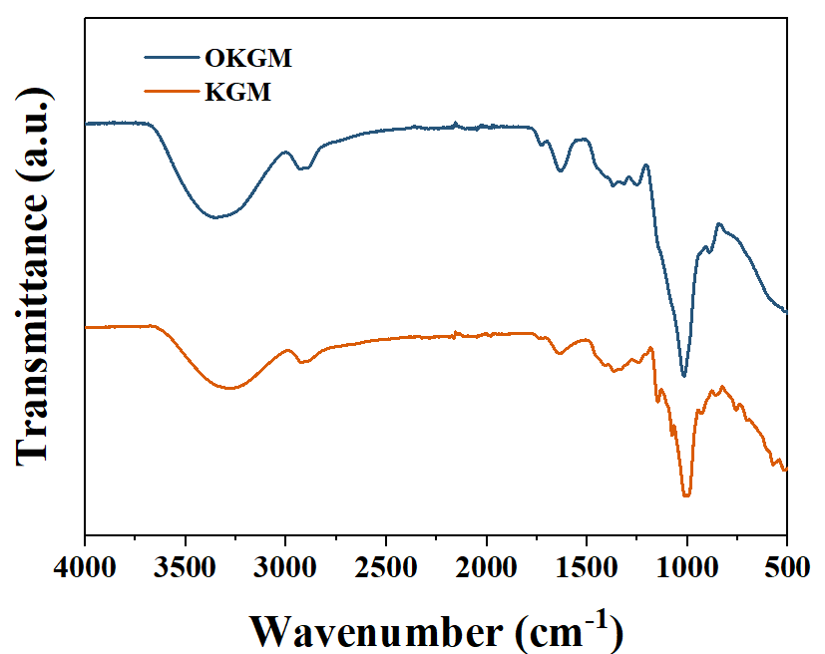
**

***Fig. S1.*** *Fourier transform infrared (FTIR)* *spectra of KGM and OKGM.*


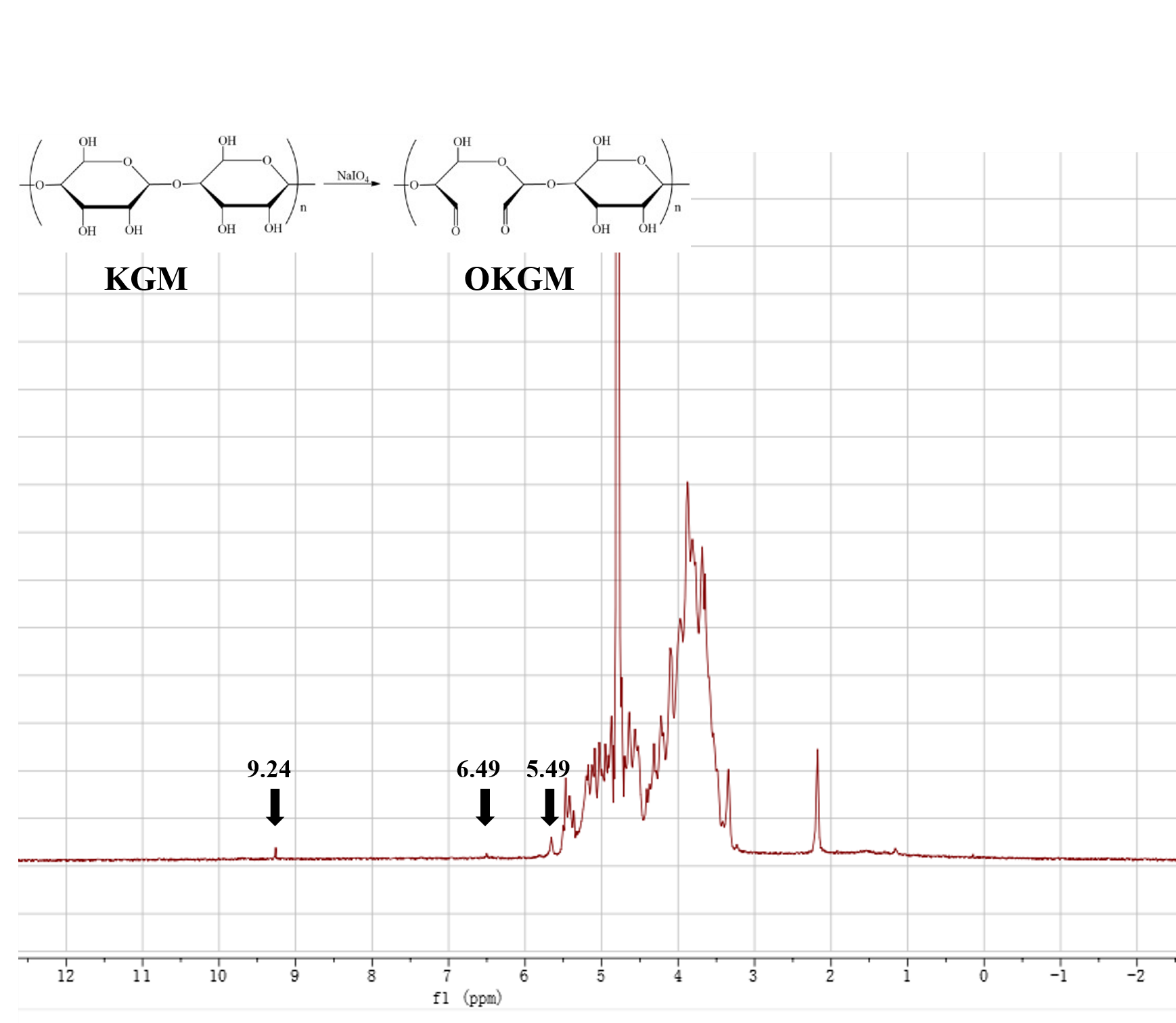


***Fig. S2.*** *^1^H NMR spectra of OKGM.*


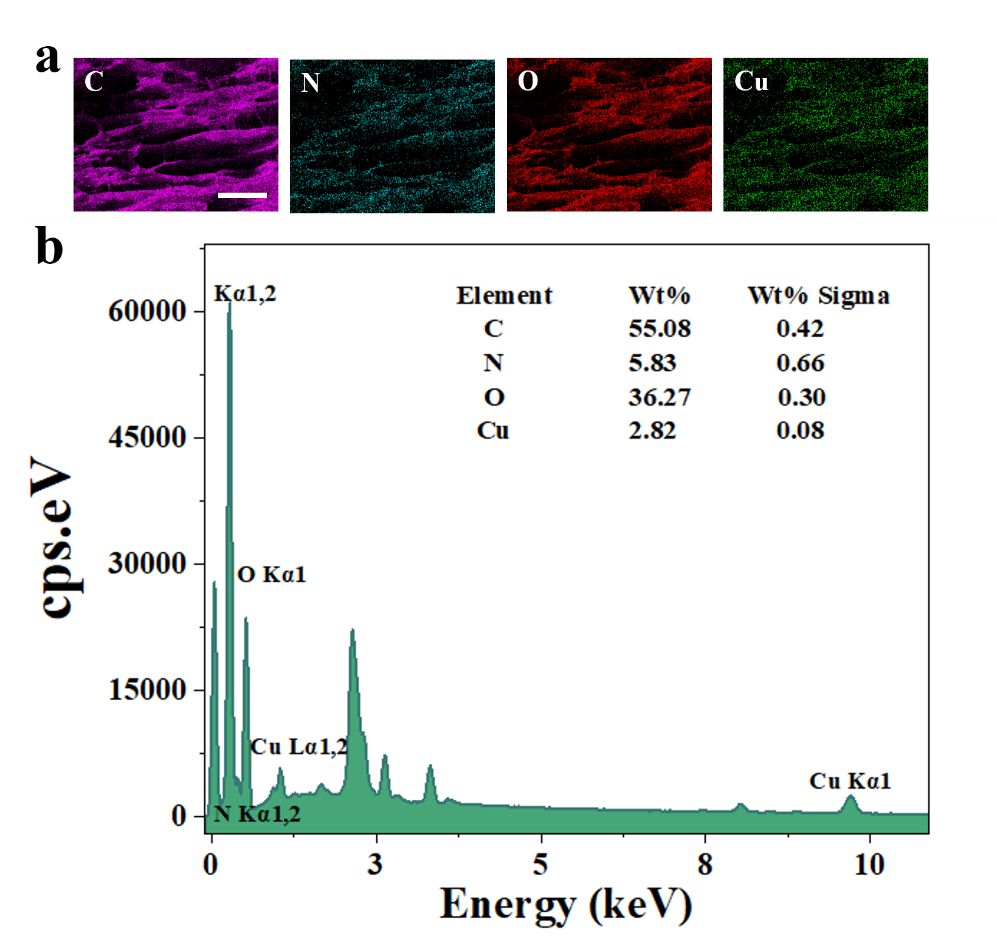


***Fig. S3.*** *Energy Dispersive spectrometer (EDS) mapping images of GEK hydrogel; scale bar is 50 μm.*


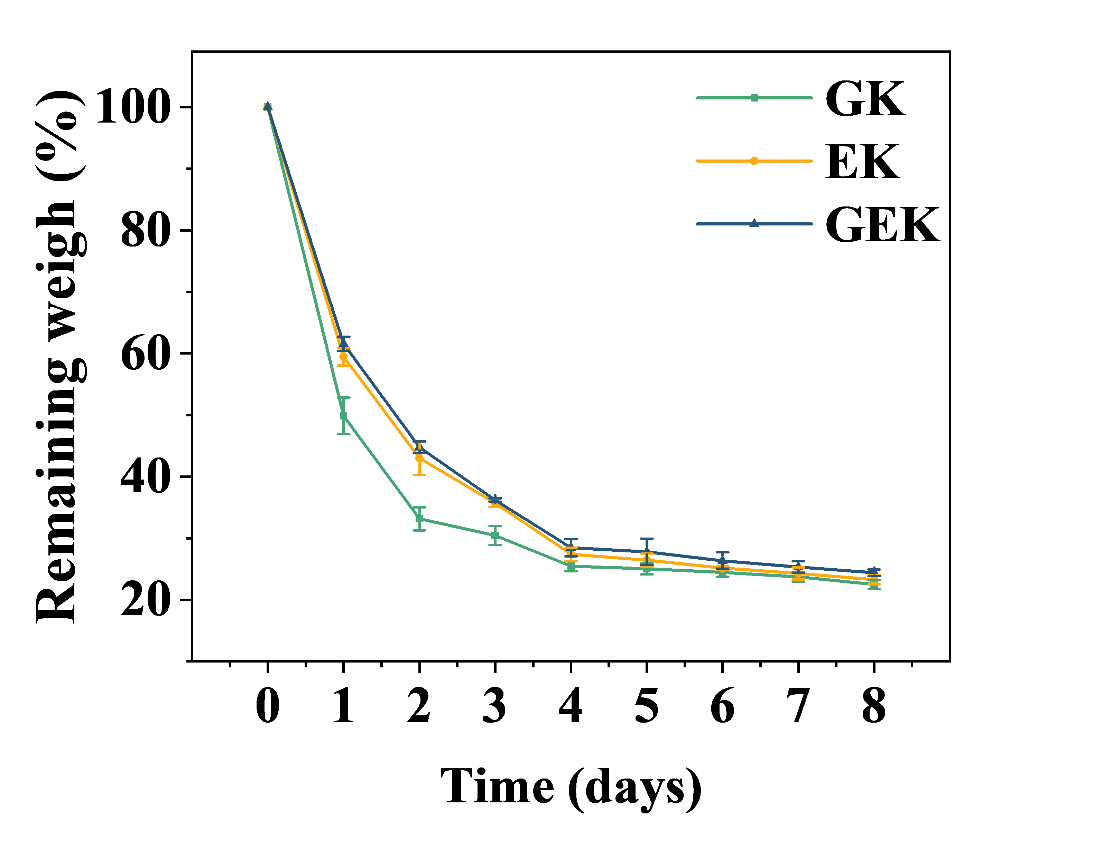


***Fig. S4.*** *In vitro degradation curve of hydrogels.*


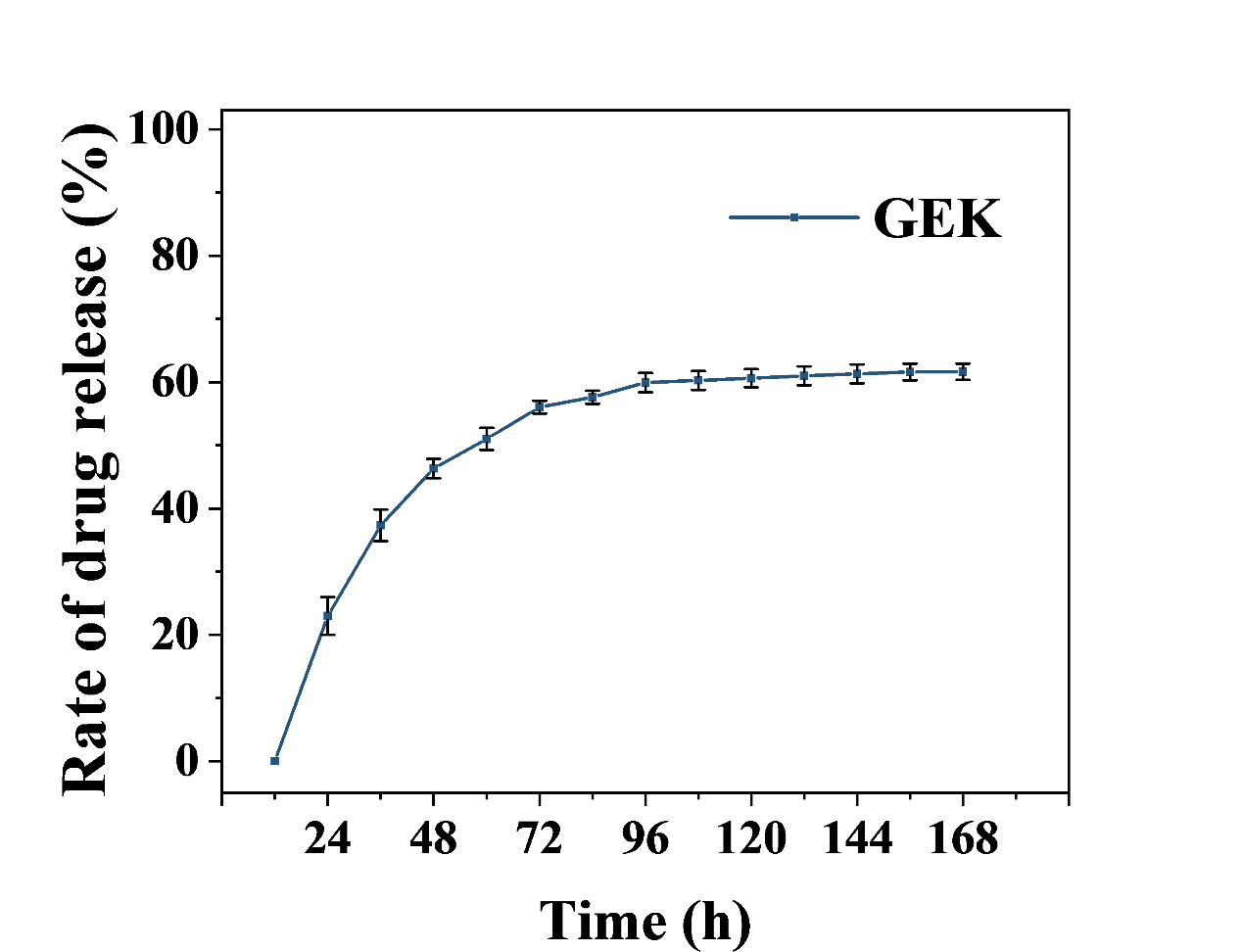


***Fig. S5.*** *Release of GHK-Cu from GEK hydrogel*


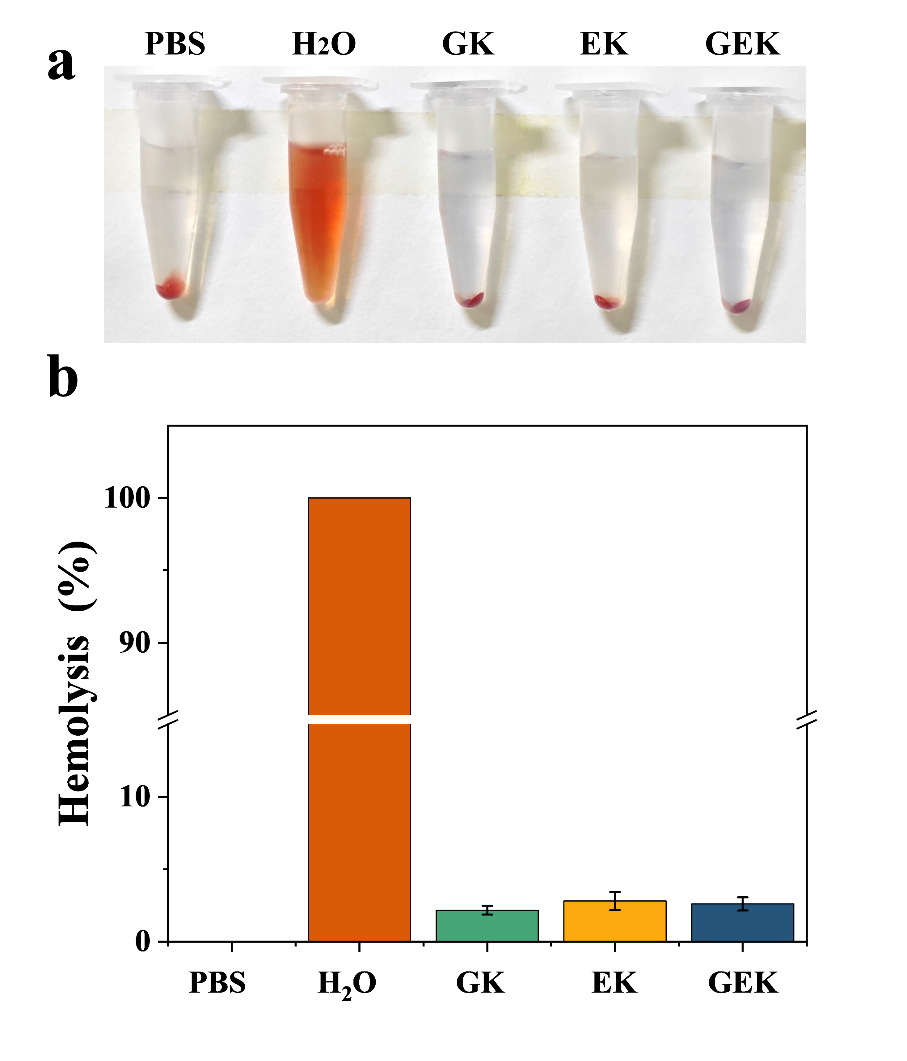


***Fig. S6.*** *Hemolysis test.*


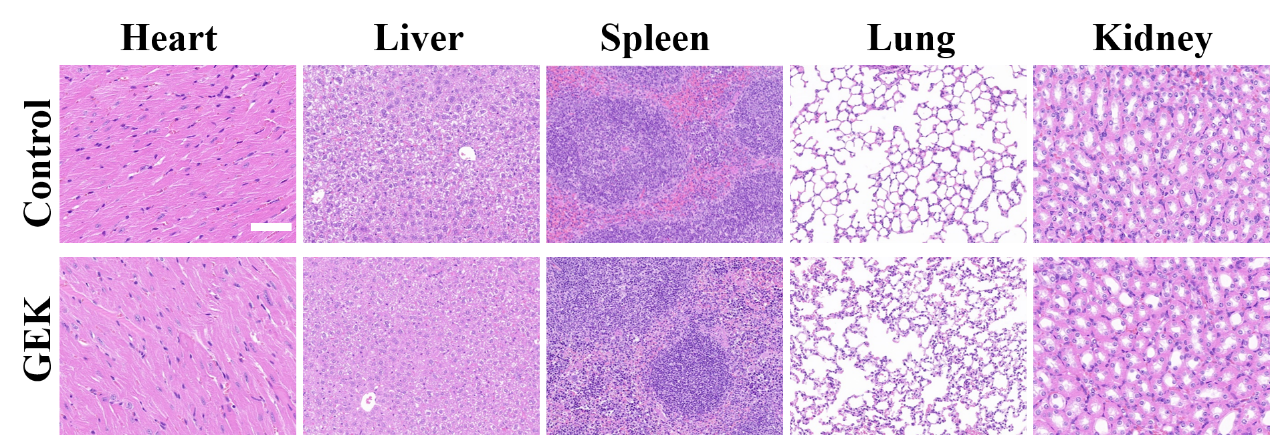


***Fig. S7.*** *Organ toxicity test, HE staining of heart, liver, spleen, lung and kidney, scale bar is 50 μm*.


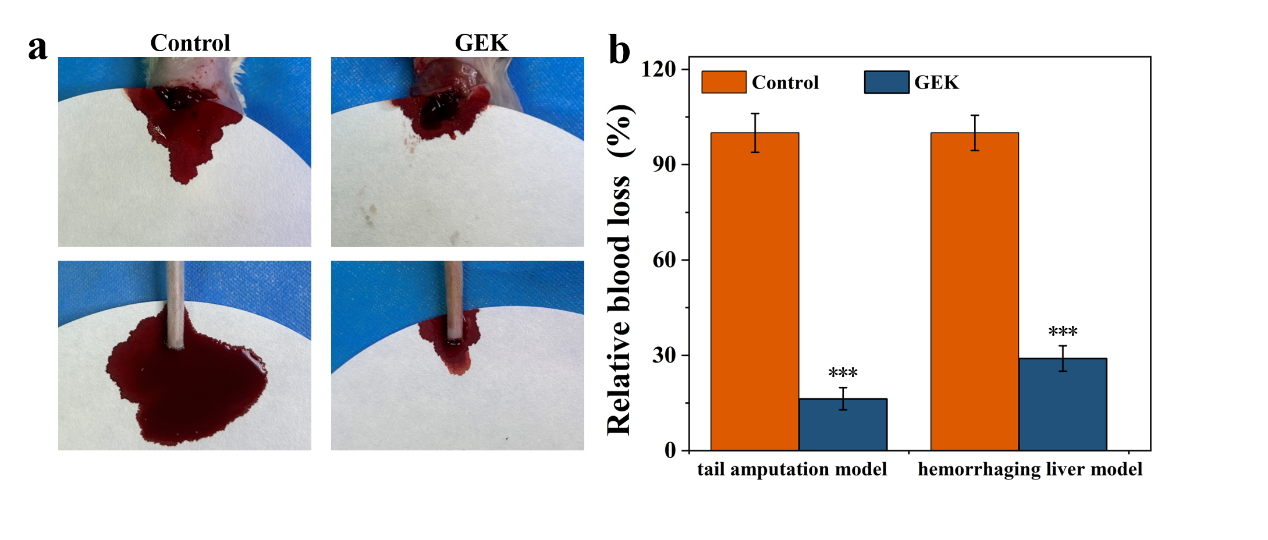
***Fig. S8.*** *Hemostatic performance test of GEK hydrogel*
